# Infidelity among parents in committed relationships during the COVID-19 pandemic

**DOI:** 10.1371/journal.pone.0329015

**Published:** 2025-08-13

**Authors:** Jessica T. Campbell, Luis F. Viegas de Moraes Leme, Amanda N. Gesselman

**Affiliations:** 1 The Center for Evaluation, Policy, and Research, Indiana University, Bloomington, Indiana, United States of America; 2 The Kinsey Institute, Indiana University, Bloomington, Indiana, United States of America; 3 University of Pennsylvania, Philadelphia, Pennsylvania, United States of America; 4 School of Nursing, Indiana University, Bloomington, Indiana, United States of America; Fakultet za pravne i poslovne studije dr Lazar Vrkatic, SERBIA

## Abstract

The COVID-19 pandemic has profoundly affected various aspects of personal life, including romantic relationships. While existing research has explored the pandemic’s impact on relationship quality and behavior, little attention has been given to the influence of the pandemic on infidelity. This study aims to explore how the COVID-19 pandemic may have influenced both the increased desire for and the actual engagement in infidelity among individuals in relationships, with a particular emphasis on parents. We conducted a cross-sectional survey of romantically partnered U.S. adults (*N *= 1,070), to examine self-reported increased desire for and engagement in infidelity. Using linear and binary logistic regressions, we investigated how parental status (parent vs. non-parent) affected this desire or engagement, with gender (men vs. women) included as a moderating variable and controlling for the occurrence of one or more stressful relationship events during the COVID-19 pandemic. Our findings indicated that parents (vs. non-parents) reported increased desire for, and engagement in, infidelity; men also reported increased desire and engagement than did women, but gender did not moderate the links between parental status and infidelity. These results suggest that parents and men may be especially vulnerable to high stress like that brought about by the COVID-19 pandemic; these individuals and their current romantic relationships may benefit from targeted social support.

## Introduction

The COVID-19 pandemic undoubtedly introduced substantial changes and challenges across multiple facets of people’s lives, including in their mental, emotional, and professional well-being, as well as their relationships with others [1,2]. At the height of the pandemic, the American Psychological Association found that 78% of American adults surveyed said that the COVID-19 pandemic was a significant source of stress, while 67% reported experiencing higher levels of stress through the course of the pandemic [3]. In general, the COVID-19 pandemic had considerable personal and social impacts, especially as it relates to connectedness; periods of lockdowns and social distancing exaggerated stress and loneliness and strained existing romantic and sexual relationships [4]. Parents in particular felt the pressure of this global event, reported feeling significantly more stressed compared to those without children [5].

While several studies have captured shifts in relationship quality or behavioral dynamics within romantic relationships [2], one relatively unexplored area is how the pandemic impacted infidelity, or the “breach of trust and/or violation of agreed upon norms” [6]. Factors resulting from the pandemic, such as potentially increased stress and strain on relationships, restrictions on social interactions and subsequent loneliness, and upheaval of daily routines likely influenced how people felt about and behaved within their relationships [4,7]. Although emerging research suggests that the impact of the pandemic on family life was not universally negative [8], some monogamous relationship partners with children may have experienced a magnification of stressors as they navigated increased childcare demands [9]. Indeed, some families had to navigate through family income or job loss, economic difficulties, worsening mental health and illness amidst the pandemic, impacting overall family wellbeing [10]. Though parents seemed to have some buffers against declines in overall marital satisfaction [11], the shifts in paid versus unpaid labor during the pandemic had negative consequences on general satisfaction levels, with mothers being especially inclined to report dissatisfaction for work-life balance [12].

For the partners experiencing more stress, the COVID-19 pandemic may have created a context in which some struggled to meet their romantic partner’s needs for emotional or sexual fulfillment, or to have their own needs met [13,14]. Tepeli Temiz and Elsharnouby (2022) found that there was a negative relationship between COVID-19 stress and relationship satisfaction, suggesting some people may have experienced heightened relational dissatisfaction or disconnection in response to external pressures [15]. Beyond the scope of the pandemic, Vowels and colleagues (2022) demonstrated that relational characteristics, including relationship satisfaction and sexual desire, were among the strongest predictors of infidelity, reinforcing the notion that people may be more inclined to seek extradyadic relationships when core relational needs are unmet [16]. Taken together, these findings suggest that when stress is high and relational interactions are less optimal, some individuals may be more inclined to seek connections outside of their romantic relationship. As such, some romantic partners may have considered having their romantic and sexual needs met elsewhere, through behaviors of infidelity (e.g., cheating, online or offline) [16]. In the current study, we assessed participants’ reports of how their increased desire for infidelity changed during the COVID-19 pandemic, whether they engaged in infidelity over the first year of the pandemic (i.e., 2020–2021), and how both parental status and gender are associated with infidelity.

### Infidelity

Most people expect romantic and sexual exclusivity from their marital or cohabitating partners [17,18]. Nonetheless, infidelity—romantic or sexual interactions with an external, or extradyadic, partner when one is in a committed monogamous relationship; also known as “cheating” [19] —is estimated to impact up to a quarter of all marriages, and tends to be even more common among cohabitating, committed, non-married partners [19–22]. According to the General Social Survey (GSS), which has tracked social behaviors of people in the U.S. since 1972, approximately 10% of married people have engaged in sexual infidelity in any given year [12% of men, 7% of women; [23–25]. The prevalence of marital infidelity is likely even higher than these GSS estimates suggest: current estimates in the United States indicate nearly half of marriages end in divorce [25,26] and infidelity is the most commonly cited reason for divorce [21].

Studies suggest that more than half of men and women who engage in sexual infidelity experience separation and/or divorce from their spouses [21,27]. The potential relationship deterioration caused by infidelity can be a heavy burden for many: people who have experienced infidelity in their relationships report poorer mental health outcomes including more instances of depression, anxiety, and PTSD [28,29]. There are also physical and sexual health-related issues that can emerge from sexual infidelity, including exposure to sexually transmitted infections [19,21]. For example, one study demonstrated that less than half of those engaging in penetrative extradyadic sex (either vaginal or anal) used a condom during their most recent extradyadic encounter [30]. For couples who maintain their relationship following infidelity, both relationship and sexual satisfaction decrease significantly, and there are heightened rates of conflict that can devolve into intimate partner violence [31–33]. There are also negative consequences of infidelity related to the broader family unit, particularly in association with children from the relationship [34,35], and perhaps further exaggerated during the COVID-19 pandemic as a byproduct of multimodal stressors (e.g., social, emotional, and financial) [34–36].

While some research has highlighted similar rates of infidelity across men and women [20,21,37,38], some literature suggests that there are gender differences in reported infidelity. Cross-culturally, men are frequently implicated as being more likely to engage in infidelity relative to women, though the form of infidelity may offer further insight into gender-based tendencies [24,37,39]. For example, acts of sexual infidelity (i.e., specifically engaging in sexual acts with an extradyadic partner, but not necessarily forming an extradyadic romantic or emotional bond) appear to be more common among men [40]. Conversely, emotional infidelity (i.e., forming an extradyadic romantic or emotional bond, but not necessarily having extradyadic sex) tends to be more common among women [40].

Beyond a sexual or emotional dichotomy, there is evidence to suggest that men engage in infidelity—broadly defined—more often than do women. This gender difference has been documented in studies of both in-person and internet-based infidelity [19,40,41]. For example, across over 16,000 participants from the year 1991–2008, women engaged in sexual infidelity at approximately half the rate of men [28]. Similarly, among a sizable sample of married men and women (*N* > 5,000), 7.13% of women engaged in infidelity, whereas 14.15% of men engaged in infidelity [42]. In sum, the amassed literature suggests that in general, men are more likely than women to experience an increased desire for infidelity or to engage in infidelity, though there are some disparities in the documented research based on the nature of infidelity [42,43].

### Infidelity and parenthood

In contrast to the large literature on gender and infidelity, there are very few studies on infidelity as a function of parenthood or parent status. Parenthood often introduces heightened stress [44], as well as shifts in physical and mental wellness [44–48]. Parenthood can also cause strain for the romantic relationship of the parent(s). For example, the birth of a child into an existing partnership often leads to a significant decline in relationship satisfaction (i.e., the first seven to eight years) [49]. Relatedly, Huebner et al. (2012) found that, among gay male parents, there was a decrease in time and energy for relationship maintenance, as well as a decrease in sexual satisfaction within the relationship [50]. These men also reported increased commitment to their relationship, however.

More recently, Lacker and colleagues (2020) studied the role of fatherhood as it related to romantic relationship outcomes [51]. Compared to non-fathers, fathers reported higher levels of relationship satisfaction as well as more instances of infidelity compared to non-fathers. In this context, Lacker et al. (2020) argued that when a child is born (through about seven or eight years of age), they require significant attention that can challenge overall relationship satisfaction, create space for unmet needs amongst men, and thus may lead to an increased risk of infidelity among fathers [51]. Although we know of no other studies on parenthood and infidelity, and the existing literature is nuanced in how relationship outcomes are impacted by parenthood, these studies suggest that the requirements of parenting may facilitate unmet emotional or sexual needs within a wide array of romantic partnerships – particularly those within the confines of a monogamous relationship.

### Infidelity and the COVID-19 pandemic

While individual differences like gender and parent status are likely important factors for relationship attitudes and behaviors in general, it is critical to consider the external context in which romantic relationships are existing. The vulnerability-stress-adaptation model (VSA) [52,53] is a theoretical framework that describes how personal and relational factors interact with stressors external to the relationship, subsequently influencing relationship (and individual) outcomes. The VSA model suggests that relationships with higher levels of vulnerability are less able to manage stressors in ways that produce positive relationship outcomes. For example, Overall et al. (2022) found that heightened external stress (i.e., mandated quarantine during the COVID-19 pandemic) and pre-existing individual vulnerabilities (i.e., attachment insecurity) amplified the risk of relationship problems, lower relationship quality, and “less cohesive family environments” over the pandemic [54].

The present study investigated romantic relationships within the context of the COVID-19 pandemic. Although research indicates that family relationships acted as a buffer in some contexts amidst the pandemic [8,55–57], many others – particularly parents – experienced heighted stress as a result of the pandemic, with mothers disproportionately impacted [58–61]. From the perspective of the VSA model, the COVID-19 pandemic acted as an external stressor for these individuals and couples, because it created an ongoing situation in which individual- and couple-level coping resources were taxed [62,63]. As a result, existing vulnerabilities that relationship partners may have had—that is, individual or couple-level characteristics that make them more susceptible to negative outcomes—were likely to be amplified.

Here, we suggest that parenthood (i.e., having children within one’s romantic relationship) could serve as one such vulnerability for some relationship partners during the COVID-19 pandemic. For such individuals, this heightened vulnerability may have facilitated unmet emotional or sexual needs within their romantic relationship, subsequently leading to an increased desire for infidelity or engagement in infidelity. As such, we predict that parents (i.e., people with children) may be more likely than non-parents to report an increased desire for infidelity and a higher likelihood of engagement in infidelity during the COVID-19 pandemic. Further, we assessed the frequency of major relationship events occurring during the pandemic (e.g., getting married, getting pregnant, altering living arrangements with or without their partner) to both acknowledge, and control for, additional major stressors that may have influenced relationship outcomes independent of the pandemic’s broader effects.

### Study overview

In the present work, we sought to understand how the pandemic may have been associated with increased desires for infidelity or engagement in infidelity. Because of the unprecedented changes and potential stress produced by the COVID-19 pandemic, we were particularly interested in whether people with children (i.e., parents) were more likely to experience increased inclinations toward infidelity than non-parents. Because the existing literature on gendered effects in infidelity is robust, we included gender in our analyses as a main effect and moderator of the link between parental status and an increased desire for, or engagement in, infidelity. Using a large, national cross-sectional sample of heterosexual, cisgender U.S. adults in committed romantic relationships, we investigated the following hypotheses:

H1: Men (vs. women) will report an increased desire for infidelity and a higher likelihood of engaging in behaviors of infidelity during the pandemic.
H2: Parents (vs. non-parents) will report an increased desire for infidelity and higher likelihood of engaging in behaviors of infidelity during the pandemic.
H3: Men who are parents will report the largest increases in desire for infidelity and the highest likelihood of engaging in behaviors of infidelity during the pandemic, compared to (1) men who are not parents and (2) women (parents and non-parents).

Last, as an exploratory aim, we investigated the extent to which increased desire for infidelity was associated with engagement in infidelity (i.e., behavioral infidelity) during the pandemic, as the high transmission rate of COVID-19 and subsequent lockdown and social distancing regulations may have constrained opportunities for in in-person infidelity behaviors, potentially shifting behaviors toward digital and/or emotional infidelity instead.

## Method

### Data collection

Data were drawn from a larger study conducted from January 1, 2021until January 31, 2021 that assessed people’s romantic and sexual lives during the COVID-19 pandemic; data were collected online via a cross-sectional survey developed by the senior/last author of this manuscript. The study was approved as exempt by the Institutional Review Board at Indiana University (Protocol # 2101388485), as it involves no more than minimal risk to participants, which means they would experience no greater risk than they would in the course of a normal day or during routine physical exams or psychological tests, according to the IU HRPP Policy regarding Exempt Research. All data were deidentified prior to the analyses, ensuring that no identifiable information was available at any point during our research process. Below we report how we determined our sample size, all data exclusions, all manipulations, and all measures in the study. No a priori power analyses were conducted, nor was this study pre-registered. Instead, the data collection goal was to sample 2,000 U.S. adults. However, a post-hoc power analysis with G*Power indicates that our sample size gives us greater than 99% statistical power to detect a medium effect (*f* = .25) in a linear regression with one dependent variable and three predictor variables.

Participants were recruited by Prodege®, a data collection company using independent opt-in Internet research panels for population-based cross-sectional surveys. Recruitment targeting was based on demographic distributions (i.e., age [between 18 and 45 years], gender, ethnicity, region, income) reflected in the most recent Current Population Survey conducted by the United States Bureau of the Census. The broader study was funded by Hearst Communications, to generate data-driven insights that could inform journalism for *Cosmopolitan* and *Esquire* magazines. As these outlets primarily target younger adult audiences, the sponsor requested that the sample be age-restricted to reflect their typical readership demographics. Therefore, participants were required to be between 18 and 45 years old. Additionally, they were required to reside in the United States and be fluent in English. Data, study materials, and study analysis code can be found at https://osf.io/bgna6/.

### Participants

The larger study included 2,000 participants. However, to answer our target research questions, we selected participants who identified their gender as men or women, who identified their sexual orientation as heterosexual, and who defined their current relationship status as a committed, exclusive relationship (monogamous); engaged; or married. Participants who identified as non-heterosexual (*n *= 277); as being single, casually dating one or more people, or consensually non-monogamous (*n *= 780); and as a gender other than man or woman (*n *= 13) were removed from the sample. Our final sample included 1,070 participants: 572 women and 498 men between the ages of 18 and 45 years of age (*M* = 33.94 years, *SD* = 6.76 years); 67.7% were married, 5.7% were engaged, and 26.6% were in a serious, exclusive relationship (monogamous). See Table 1 for complete participant demographics.

**Table 1:** Sample demographics.

		Target subgroups			
Demographic variable	Overall sample (*N* = 1,070)	Women (*n* = 572)	Men (*n* = 498)	Parents (*n* = 771)	Non-parents (*n* = 299)
Age					
* M*	33.94 years	33.70 years	34.21 years	35.53 years	29.83 years
* SD*	6.76 years	6.66 years	6.87 years	5.75 years	7.42 years
Relationship length					
* M*	8.72 years	9.24 years	8.14 years	10.38 years	4.46 years
* SD*	6.42 years	6.59 years	6.17 years	6.32 years	4.39 years
Gender					
* *Women	53.5%	100%	—	51.1%	59.5%
* *Men	46.5%	—	100%	48.9%	40.5%
Race/ ethnicity					
* *Black/ African American	8.1%	10.3%	5.6%	6.9%	11.4%
* *East Asian	2.9%	4.5%	1.0%	2.1%	5.0%
* *Hispanic or Latino	16.2%	20.5%	11.2%	14.1%	21.4%
* *North American Indian/Alaska Native/Pacific Islander	0.9%	1.0%	0.8%	0.8%	1.3%
* *South Asian	1.6%	1.7%	1.4%	1.4%	2.0%
* *White	69.8%	61.5%	79.3%	74.2%	58.5%
* *Another identity not listed	0.5%	0.3%	0.6%	0.5%	0.3%
Annual household income					
* *Less than $15,000	7.8%	7.9%	7.6%	6.0%	12.4%
* *$15,000 to $29,999	10.7%	12.4%	8.6%	7.7%	18.4%
* *$30,000 to $44,999	12.0%	14.3%	9.2%	9.6%	18.1%
* *$45,000 to $59,999	11.9%	15.4%	7.8%	9.5%	18.1%
* *$60,000 to $74,999	12.1%	13.8%	10.0%	11.9%	12.4%
* *$75,000 to $99,999	16.4%	17.0%	15.9%	19.6%	8.4%
* *$100,000 to $149,999	17.4%	14.0%	21.3%	20.8%	8.7%
* *$150,000 or more	11.9%	5.2%	19.5%	15.0%	3.7%
Relationship status					
* *In a committed relationship	26.6%	29.7%	23.1%	13.6%	60.2%
* *Engaged	5.7%	5.1%	6.4%	4.3%	9.4%
* *Married	67.7%	65.2%	70.5%	82.1%	30.4%
Parental status					
* *Has children	72.1%	68.9%	75.7%	100%	—
* *Does not have children	27.9%	31.1%	24.3%	—	100%
Living arrangement					
* *Lives with romantic partner	85.3%	85.8%	84.7%	92.6%	66.6%
* *Does not live with romantic partner	14.8%	14.2%	15.3%	7.4%	33.4%

### Measures

Note that for many of our measures, we asked participants to report their behaviors or feelings “now compared to before the pandemic.” In the survey, we defined ‘before the pandemic’ as the year prior to the start of the pandemic.

#### Demographics.

Participants self-reported their demographics, including age, gender identity, sexual orientation, race/ethnicity, current relationship status, parental status, and current employment status.

#### Parental status.

Participants reported whether or not they were parents by responding to the question, “Do you have children?” Participants responded on a binary scale where 1 = *yes* and 2 = *no*. A large portion of the sample (72.1%; *n *= 771) reported being a parent.

#### Engagement in infidelity during the pandemic.

Participants reported their engagement in infidelity by responding to the question, “Since the pandemic began, have you engaged in anything your partner would consider infidelity?” Participants responded with one of the following options: 1 = *Yes, I’ve done something in real life (i.e., not online)*, 2 = *Yes, I’ve done something online or via messages but not in-person*, 3 = *Yes, I’ve done something both online and in person,* 4 = *I’m not sure*, 5 = *I don’t know* and 6 = *No.* Note that this item was intentionally phrased in terms of what the participant believed their partner would consider infidelity, rather than applying a universal definition, to better reflect the subjective and relationally-defined nature of infidelity perceptions [64,65].

#### Increased desire for sexual infidelity.

Participants were asked: “Compared to before the pandemic, how likely are you to pursue other sex partners without informing your current partner?” Participants responded on a scale where 1 = *not at all likely*, 4 = *just as likely*, and 7 = *very likely*. Higher scores represented a stronger desire to engage in sexual infidelity at the time of the survey compared to before the pandemic—in other words, a perceived increase in desire.

#### Increased desire for emotional/romantic infidelity.

Participants were asked: “Compared to before the pandemic, how likely are you to pursue other romantic relationship partners without informing your current partner?” Participants responded on a scale where 1 = *not at all likely*, 4 = *just as likely*, and 7 = *very likely*. Higher scores represented a stronger desire to engage in romantic infidelity at the time of the survey compared to before the pandemic—in other words, a perceived increase in desire.

#### Occurrence of other major relationship events during the pandemic.

Participants were asked, “Did you experience any of the following relationship events during the pandemic?” The list included the following options: *I started dating someone new; I entered into a committed relationship with a new partner; I got back into a relationship with an ex-partner; I entered into an open relationship; I entered into a consensually non-monogamous relationship; I broke up with someone I was in a relationship with; I was broken up with (e.g., ‘dumped’) by a relationship partner; I became engaged; I got married; I got divorced or separated; My partner and I broke up/ separated and then got back together; I moved in with a relationship partner; I moved out of a place I shared with a relationship partner, we no longer live together; I got pregnant (or my partner did); I cheated on my partner (ex or current); my partner cheated on me (ex or current);* or *something else*.

### Data analysis plan

The IBM® SPSS® Statistics version 29.0.2.0 software program was used to analyze the data. First, to explore engagement in infidelity, we created a binary variable comparing those who reported engaging in any form of infidelity (i.e., online or in-person, coded as 1) to those who reported not engaging in any forms of infidelity (i.e., those who selected ‘no’ for this item; coded as 0). Participants who selected ‘I don’t know’ or ‘I’m not sure’ were recoded as missing. Second, after observing a strong correlation between increased desire for sexual infidelity and increased desire for emotional/romantic infidelity (*r* = .84, *p* < .001), we opted to combine these two variables into one by averaging them. As such, all analyses assessed the increased desire for infidelity in general rather than an increased desire for sexual or emotional/romantic infidelity.

In terms of analyses, we first conducted descriptive statistics for increased desire for infidelity and engaging in infidelity, examining percentages and means for the overall sample. Following this, we conducted hierarchical regression models to examine whether gender and parental status meaningfully interacted to predict increased desire for infidelity. This included a linear regression model predicting the increased desire for infidelity, and one logistic regression model predicting infidelity engagement. In both models, parental status (−1 = non-parents, 1 = parents), gender (−1 = women, 1 = men), and their two-way interaction term were entered as predictor variables. These models also included three covariates to allow us to control for their effects: participant age, relationship length and a binary variable indicating whether participants had experienced any of the assessed major relationship events. In this latter variable, participants who had experienced none of the assessed relationship events were coded as 0 (33.9%), while participants who had experienced one or more of those major events was coded as 1 (66.1%). Note that both the linear and binary logistic regression models were hierarchical, with predictor variables and covariates entered on Block 1, and the interaction term entered on Block 2. If the interaction terms were not significant, they were dropped from the model and results from Block 1 were interpreted. Additionally, for the linear regression model, we assessed regression assumptions by examining residual normality (via skewness and kurtosis values, the Shapiro-Wilk test, and visual inspection of the histogram and Q-Q plot) and multicollinearity (using Variance Inflation Factor [VIF] values).

Finally, we conducted a separate binary logistic regression to investigate our exploratory aim – whether increased desire for infidelity predicted engagement in infidelity. This model included engagement in infidelity as the outcome variable and included gender and parental status as predictor variables. However, this model also included increased desire for infidelity as a predictor variable. In all analyses, statistical significance was determined by *p*-values less than 0.05.

## Results

### Pandemic-influenced desires for and engagement in infidelity

On average, participants reported that they were relatively unlikely to pursue other romantic or sexual partners compared to how they felt prior to the pandemic (*M* = 2.38, *SD* = 1.87). In particular, only 19.6% (95% CI[16.9%, 22.3%]) of participants selected a response option above the scale’s midpoint (i.e., above *‘just as likely*’), indicating that they were now more likely to pursue extradyadic partners. Looking within subgroups, the following selected a response above the scale’s midpoint: 29.2% of men (95% CI [24.8%, 33.6%]), 9.7% of women (95% CI [6.7%, 12.6%]), 24.2% of parents (95% CI [20.7%, 27.7%]), and 8.3% of non-parents (95% CI [4.7%, 11.8%]).

Conversely, 71.0% (95% CI [67.9%, 74.2%]) of the overall sample selected a response option below the scale’s midpoint, indicating that they were now less likely to pursue extradyadic partners. In terms of subgroup responses, 60.5% of men (95% CI [55.8%, 65.3%]), 81.9% of women (95% CI [78.1%, 85.7%]), 67.1% of parents (95% CI [63.2%, 70.9%]), and 80.9% of non-parents (95% CI [75.8%, 86.0%]) selected a response option above the scale’s midpoint.

Next, 18.8% (95% CI [16.4%, 21.2%]) of participants reported engaging in something during the pandemic that their partner would consider to be infidelity. This includes 28.1% of men (95% CI [24.0%, 32.1%]), 10.9% of women (95% CI [8.3%, 13.5%]), 20.7% of parents (95% CI [17.8%, 23.6%]), and 13.9% of non-parents (95% CI [9.9%, 17.9%]) who engaged in infidelity.

### Increased desire for infidelity at the intersection of gender and parental status

We conducted a linear regression model predicting increased desire for infidelity, with gender, parental status, and their two-way interaction term entered as predictor variables along with age, relationship length, and whether participants had experienced a major relationship event entered as control variables. To evaluate the assumptions of our linear regression analysis, we conducted a series of diagnostic tests. Skewness and kurtosis values for the standardized residuals were within acceptable bounds (i.e., 0.78 and −0.34, respectively), suggesting no substantial departures from normality in the residual distribution. While the Shapiro-Wilk test for normality was statistically significant (p < .05), such significance is common in large samples and does not necessarily indicate meaningful non-normality. Visual inspection of the Q-Q plot and histogram of residuals indicated an approximately normal distribution. Additionally, multicollinearity diagnostics showed that all predictors had Variance Inflation Factor (VIF) values below 5.0 (i.e., between 1.21 and 1.69), indicating no concerns regarding multicollinearity.

Results from the hierarchical linear regression model showed no significant interaction between gender and parental status, illustrating a lack of support for hypothesis 3. As such, we dropped the interaction from the model, and interpreted results from Block 1, which contained only the predictor and control variables. The overall model was statistically significant, *F*(5, 795) = 30.65, *p* = < .001, *R*^2^ = 0.156. The model explained approximately 16.2% of the variance in increased desire for infidelity. Additionally, found significant main effects for gender and parental status (see Table 2). Men were more likely to demonstrate an increased desire for infidelity compared to women, and parents were more likely to express an increased desire for infidelity compared to non-parents. All three control variables were significant in the model as well, with older (vs. younger) age, shorter (vs. longer) relationships, and participants who did (vs. did not) experience a major relationship event reporting an increased desire for infidelity.

**Table 2:** Coefficients from the linear regression model predicting increased desire for infidelity.

Predictor	*B*	*SE*	β	*p*	95% CI_B_	*r*_p_
Intercept	0.696	0.349	—	0.046	0.011,1.381	—
Age	0.048	0.011	0.175	<.001	0.027, 0.069	0.155
Relationship length	−0.004	0.001	−0.144	<.001	−0.006, −0.002	−0.111
Experienced major relationship event (no/yes)^a^	0.691	0.138	0.180	<.001	0.421, 0.961	0.175
Gender (women/men)^b^	0.428	0.062	0.229	<.001	0.306, 0.549	0.238
Parental status (non-parent/parent)^c^	0.333	0.076	0.161	<.001	0.184, 0.482	0.154

*Note*. The outcome variable is increased desire for infidelity (continuous).

^a^Coded 0 = did not experience any major relationship events; 1 = did experience a major relationship event.

^b^Coded −1 = women, 1 = men.

^c^Coded −1 = non-parents, 1 = parents.

### Engagement in infidelity at the intersection of gender and parental status

Results from the hierarchical binary logistic regression model showed no significant interaction between gender and parental status, illustrating a lack of support for hypothesis 3. As such, we dropped the interaction from the model, and interpreted results from Block 1, which contained only the predictor and control variables. The model was statistically significant, χ^2^(5) = 176.30, *p* < .001, indicating that the predictors reliably distinguished between those who had and had not engaged in infidelity. The model explained 25.5% of the variance in infidelity engagement (Nagelkerke *R*^2^ = .255) and correctly classified 83.7% of cases. There were significant main effects for gender and parental status (see Table 3). Regarding gender, the odds of engaging in infidelity were 70% higher for men than for women (*p* < .001). Regarding parental status, parents’ odds of engaging in infidelity were 48% higher than odds for non-parents. All three control variables significantly predicted engagement in infidelity. Older (vs. younger) age, shorter (vs. longer) relationship length, and experiencing (vs. not experiencing) a major relationship event were all associated with higher odds of engaging in infidelity.

**Table 3:** Coefficients from the binary logistic regression model predicting engagement in infidelity.

Variable	OR	95% CI	*p*
Intercept	0.031	—	<.001
Age	1.040	1.009, 1.072	.011
Relationship length	−0.004	0.993, 0.999	.010
Experienced major relationship event (no/yes)^a^	5.920	4.043, 8.668	<.001
Gender (women/men)^b^	1.700	1.419, 2.036	<.001
Parental status (non-parent/parent)^c^	1.479	1.178, 1.858	<.001

*Note.* The outcome variable is engagement in infidelity (0 = no, 1 = yes).

^a^Coded 0 = did not experience any major relationship events; 1 = did experience a major relationship event.

^b^Coded −1 = women, 1 = men.

^c^Coded −1 = non-parents, 1 = parents.

### Exploratory aim: Does desire predict behavior?

Finally, our exploratory analyses showed that engagement in infidelity was significantly, positively predicted by increased desire for infidelity (see Table 4). The model was statistically significant, χ^2^ (6) = 271.90, p < .001, indicating that the predictors reliably distinguished between those who had and had not engaged in behaviors of infidelity. The model explained 44.6% of the variance in infidelity engagement (Nagelkerke R^2^ = .446) and correctly classified 83.0% of cases. Participants who reported an increased desire for infidelity over the pandemic were more likely to have engaged in infidelity over that timeframe, compared to participants with a weaker desire for infidelity. In this model, with differences in increased desire for infidelity accounted for, gender, relationship length, and age were not significant predictors of engagement in infidelity. Parental status did emerge as a significant main effect, with parents having 33% higher odds of engaging in infidelity compared to non-parents’ odds. Likewise, participants who had experienced a major relationship event had 393% higher odds of engaging in infidelity compared to participants who had not experienced a major relationship event.

**Table 4:** Coefficients from the binary logistic regression undertaken for the exploratory aim of assessing whether increased desire for infidelity predicted engagement in infidelity.

Variable	OR	95% CI	*p*
Intercept	0.098	—	<.001
Age (mean-centered)	1.012	0.976, 1.050	.504
Relationship length (mean-centered)	0.999	0.995, 1.002	.416
Experienced major relationship event (no/yes)^a^	4.926	3.115, 7.790	<.001
Gender (women/men)^b^	1.172	0.941, 1.460	.156
Parental status (non-parent/parent)^c^	1.330	1.015, 1.742	.038
Increased desire for infidelity (mean-centered)	1.853	1.651, 2.079	<.001

*Note.* The outcome variable is engagement in infidelity (0 = no, 1 = yes).

^a^Coded 0 = did not experience any major relationship events; 1 = did experience a major relationship event.

^b^Coded −1 = women, 1 = men.

^c^Coded −1 = non-parents, 1 = parents.

## Discussion

The present study explores the complex dynamics of infidelity within the framework of the COVID-19 pandemic, a period marked by unprecedented societal and personal challenges. With guidance from the Vulnerability-Stress-Adaptation (VSA) model, we sought to understand how heightened external stressors—like those introduced by the pandemic—intersect with individual and relational vulnerabilities like parental status. Our findings contribute to the literature by delineating the nuanced ways in which the COVID-19 pandemic may have shaped increased desire for infidelity and engagement in infidelity, particularly among parents. This analysis not only highlights the critical role of external stressors in influencing relationship dynamics but also provides foundation for future research into infidelity during the pandemic.

Despite the significant disruptions caused by the COVID-19 pandemic, our findings revealed that the overall increased desire for infidelity among participants was relatively low, with an average of 2.38 on a 7-point scale. Notably, only 19% of respondents reported an increase in their increased desire for infidelity (i.e., answered above the scale midpoint), suggesting that the pandemic did not universally intensify infidelity inclinations. Concurrently, our data indicated that 19% of participants had engaged in some form of infidelity during the pandemic, with activities split between online and in-person interactions. This distribution underscores the complex role of social distancing measures, which were intended to limit physical contact. Nearly half of those engaging in infidelity during the pandemic had done so in person, suggesting a lack of adherence to social distancing measures. However, these distancing measures may have deterred some from seeking in-person extradyadic interactions, motivating nearly half of these individuals to seek out such extradyadic interaction online. These results may reflect an adaptation to the imposed constraints and a possible shift in the modalities through which infidelity commonly occurs.

Additionally, results from the current study offered support for the two of the three hypotheses. Foremost, men reported higher levels of increased desire for, and engagement in, infidelity during the COVID-19 pandemic relative to women (supporting H1). These findings are consistent with existing literature demonstrating gender differences in infidelity, particularly highlighting that men are more likely to desire or engage in infidelity relative to women. Various theoretical explanations exist for why men may find infidelity more appealing, including evolutionary perspectives on paternal investment and societal ‘sexual double standards’ that punish women for engaging in casual sex [66–70]. Within the framework of the VSA, these gender differences may be understood as differential vulnerabilities that are exacerbated by pandemic-related stressors, leading to varying adaptations in how men and women navigate their stressors with regards to infidelity [52,54].

The dynamics of infidelity also varied significantly when considering parental status, which can be contextualized within the VSA. Parents reported a moderately higher increased desire for infidelity and higher odds of engaging in infidelity during the COVID-19 pandemic compared to non-parents (supporting H2). This suggests that the stress and challenges associated with parenting might heighten vulnerabilities and increased desire for extradyadic interactions, and that these desires may indeed manifest into actions. This highlights how parental responsibilities, under the strain of a global crisis, can exacerbate the stress-response behaviors predicted by the VSA model, emphasizing the complex interplay between individual vulnerabilities, stress, and adaptive processes in romantic relationships. These results are also consistent with prior research that suggests that heightened stress, particularly during the COVID-19 pandemic, can diminish relationship satisfaction [14,15] and that unmet sexual and romantic needs tend to be robust predictors of infidelity [16].

Beyond an examination of main effects, we conducted further analyses to understand interactions between gender and parental status in relation to desires and behaviors of infidelity. While we hypothesized that men who were parents would exhibit the highest levels of both desire and actual engagement in infidelity, the results did not support any significant interaction effects (H3 unsupported). Both mothers and fathers in our sample displayed similar patterns of increased desire for and engagement in infidelity. This lack of significant interaction suggests that the impact of the pandemic on infidelity may be more widely experienced among genders, rather than being distinctly heightened among fathers. Our findings invite a re-evaluation of traditional assumptions about gender roles within the context of relationship stressors brought on by external crises such as the COVID-19 pandemic.

Further analysis of covariates in our regression models revealed insights with regards to age and the experience of stressful relationship events during the pandemic. Age emerged as a significant predictor of the increased desire for infidelity, with older participants reporting a greater inclination towards infidelity than younger participants. This could suggest that longer durations in relationships or accumulated life stress might impact relationship satisfaction, sexual satisfaction, or fidelity. Additionally, individuals who experienced major relationship events during the pandemic—such as getting engaged, married, or experiencing a breakup—reported higher desires for infidelity and higher odds of engaging in infidelity, compared to participants who did not experience a major relationship event during the pandemic. According to the VSA, these findings can be interpreted as indicators of how specific life events and age-related factors serve as vulnerabilities. Major life events might exacerbate stress and vulnerabilities in relationships, increasing the propensity towards infidelity in response to pandemic-induced stressors.

Finally, our study also explored the relationship between the increased desire for infidelity and actual engagement in infidelity during the COVID-19 pandemic. The results revealed a positive association between these two variables. Individuals who reported a higher increased desire for infidelity were more likely to have engaged in infidelity during the pandemic. This relationship suggests that the pandemic may have heightened feelings of discontent or restlessness within romantic relationships, which in turn led to an increased likelihood of acting on these desires. This finding aligns with the VSA, which posits that external stressors can amplify pre-existing vulnerabilities and shape the coping mechanisms that individuals employ. In this context, the increased desire for infidelity, intensified by the stress of the pandemic, appears to have translated into actual extradyadic activities despite potential barriers such as limited social opportunities due to lockdowns and social distancing measures.

### Future directions

The current study provides data from a large, national sample of American adults that were surveyed during the first year of the COVID-19 pandemic and offers a snapshot of a unique period in history in which stress was significantly heightened—particularly among parents. However, the current research is not without its limitations. A key limitation of this study is that we did not directly measure perceived stress, making it impossible to determine whether pandemic-related distress universally contributed to increased desires for infidelity or engagement in infidelity. According to modern stress theories, stress is not simply a function of external events but rather of how individuals cognitively appraise those events [69]. Some individuals may have experienced heightened relationship stress due to increased childcare responsibilities and financial uncertainty, while others may have perceived the pandemic as an opportunity for increased family bonding and a rebalancing of work-life responsibilities [55–62]. Future research should incorporate validated measures of perceived stress and examine how differences in stress appraisal influence infidelity-related attitude and behaviors. By doing so, researchers can better distinguish between cases in which infidelity is a response to external stressors versus other relational or individual factors.

Other methodological limitations also exist in our study. First, data were collected at a single time point, preventing deeper probing of long-term consequences of heightened stress on fidelity in monogamous, heterosexual relationships. Notably, the cross-sectional nature of the present research restricted the ability to determine causality or to glean deeper insight into the downstream influences that increased desire for, or behaviors of infidelity had on couples in the context of the COVID-19 pandemic and beyond.

Second, the data were based on self-reported responses. Discussing infidelity is potentially sensitive, particularly if those who have engaged in infidelity have not addressed their behaviors with their current romantic partner. Thus, participants may have withheld or under-reported desires for, or engagement in, infidelity. Third, we did not collect data on the number of children that participants had, the ages of those children, or whether participants had full custody of those children (i.e., whether the children lived in the participant’s home full-time). The research also lacks more nuanced information regarding relationship or marital duration, which would elucidate patterns related to how relationship length may influence inclinations toward infidelity. Integrating these components in future research would allow for a deeper understanding of how relational dynamics further inform increased desires and behaviors of infidelity.

Additionally, our survey items were designed to assess perceived change in desire for infidelity relative to the period before the pandemic, rather than absolute levels of likelihood. The response scale was centered at 4 (“just as likely”), with lower and higher scores indicating a perceived decrease or increase, respectively. While baseline desire is not directly measured, this format allowed us to capture subjective appraisals of change, which is especially relevant in the context of a major life disruption like the COVID-19 pandemic. However, we did not have pre-pandemic data on desires for infidelity or engagement in infidelity, which would allow for more accurate analyses of change over time as a byproduct of the COVID-19 pandemic. Future researchers should collect more extensive detail and make use of longitudinal methodologies to further illustrate the association between the COVID-19 pandemic and related stress on infidelity.

Moreover, the current research primarily relies on single-item measures of participants’ desires for, and engagement in, behaviors of infidelity. Relatively recent research associated with single-item measures acknowledge the general validity and reliability of such items compared to multi-item measures, especially in the context of psychological science [71,72]. Single-item measures are especially well suited for topics that are clearly and narrowly defined, and such measures have value in minimizing participant burden [72,73]. However, single-item measures do not capture depth or nuance related to participants’ desires and behaviors, thereby potentially limiting the broader generalizability of the present findings. Future research should incorporate multi-item measures and qualitative assessments of infidelity among parents in monogamous relationships. Measures that are equipped to capture the depth and nuance related to infidelity (e.g., motivations, sexual infidelity, emotional infidelity, and level of emotional involvement) should be prioritized in any subsequent research.

Further, our research prioritized heterosexual participants due to limited sample sizes of participants who identified as something other than heterosexual (i.e., gay, lesbian, or bisexual) and met the additional inclusion criteria. Future research should prioritize the experiences of gay, lesbian, and bisexual couples in the realm of infidelity, both within the context of the COVID-19 pandemic, other major external stressors, and beyond.

Importantly, the relationship between desire and behavior is tenuous, and the presence of a desire does not always determine outcomes through action [74]. In fact, contemporary research suggests that there is only a weak to moderate correlation between desires and actual behaviors (e.g.,.24 −.54) [74–76]. The attitudes discussed here, beyond the scope of actual behaviors of infidelity, do not necessarily equate to behaviors related to infidelity.

More broadly, future research should also collect more in-depth information about the experiences of the COVID-19 pandemic and its consequences on romantic partnerships. In particular, a qualitative assessment is needed to identify the ways in which people experienced change in their relationships and whether/how they experienced unmet needs. Such work would provide a foundation for better tuning into the relationship struggles created by the COVID-19 pandemic, especially for parents. Lastly, it would also be interesting for future studies to further explore how distinct types of infidelity (i.e., emotional, sexual) can impact relationship outcomes.

## Conclusion

Infidelity, often referred to as “cheating,” carries numerous negative consequences for individuals and their relationships. However, external stressors, such as the COVID-19 pandemic, can exacerbate unmet emotional or sexual needs within monogamous relationships. Our study revealed that such stressors could heighten desires for infidelity and, in some cases, lead to acts of infidelity. Notably, we found that parenthood introduced additional complexities; parents were more likely to desire and engage in infidelity compared to non-parents. This suggests that the demands of parenting can also heighten vulnerabilities that lead to infidelity. These findings highlight the specific challenges that parents face in maintaining romantic satisfaction and stability during crises. Our results illustrate the need for targeted therapeutic interventions to support parents as they navigate their unique relationship challenges during times of immense external stress.

### Policy implications

The present findings offer several implications for relational and familial policy, particularly in the context of widespread external stress, such as the COVID-19 pandemic. Foremost, parents and caregivers may benefit from expanded access to relationship resources to support and nourish partnerships while accounting for demands of caregiving. Policies that support funding for accessible couples’ counseling, stress management, and robust flexible parenting support sources (e.g., affordable childcare, mental health leave) may help to mitigate some of the relational strain addressed in the current research. Further, family-centered interventions should extend to promote emotional and romantic well-being among the parental unit rather than solely prioritizing independent caretaking and child outcomes. As identified considerably through the course of the COVID-19 pandemic, supporting parents both as caregivers and as independent individuals in romantic partnerships is necessary to support long-term relationship and familial stability.
